# Fuzzy Gain-Scheduling PID for UAV Position and Altitude Controllers

**DOI:** 10.3390/s22062173

**Published:** 2022-03-10

**Authors:** Aurelio G. Melo, Fabio A.A. Andrade, Ihannah P. Guedes, Guilherme F. Carvalho, Alessandro R.L. Zachi, Milena F. Pinto

**Affiliations:** 1 Department of Electrical Engineering, Federal University of Juiz de Fora, Juiz de Fora 36036-900, Brazil; aurelio.melo@engenharia.ufjf.br; 2 Department of Microsystems, Faculty of Technology, Natural Sciences and Maritime Sciences, University of South-Eastern Norway (USN), 3184 Borre, Norway; 3 NORCE Norwegian Research Centre, 5838 Bergen, Norway; 4 Department of Electronics Engineering, Federal Center for Technological Education of Rio de Janeiro, Rio de Janeiro 20271-110, Brazil; ihannah.guedes@aluno.cefet-rj.br (I.P.G.); guilherme.carvalho@aluno.cefet-rj.br (G.F.C.); alessandro.zachi@cefet-rj.br (A.R.L.Z.); milena.pinto@cefet-rj.br (M.F.P.)

**Keywords:** gain schedule technique, fuzzy-PID strategy, position controller, altitude controller, UAV

## Abstract

Unmanned aerial vehicle (UAV) applications have evolved to a wide range of fields in the last decade. One of the main challenges in autonomous tasks is the UAV stability during maneuvers. Thus, attitude and position control play a crucial role in stabilizing the vehicle in the desired orientation and path. Many control techniques have been developed for this. However, proportional integral derivative (PID) controllers are often used due their structure and efficiency. Despite PID’s good performance, different requirements may be present at different mission stages. The main contribution of this research work is the development of a novel strategy based on a fuzzy-gain scheduling mechanism to adjust the PID controller to stabilize both position and altitude. This control strategy must be effective, simple, and robust to uncertainties and external disturbances. The Robot Operating System (ROS) integrates the proposed system and the flight control unit. The obtained results showed that the proposed approach was successfully applied to the trajectory tracking and revealed a good performance compared to conventional PID and in the presence of noises. In the tests, the position controller was only affected when the altitude error was higher, with an error of 2% lower.

## 1. Introduction

Unmanned aerial vehicles (UAVs) give new opportunities to the industry due to their flexibility of flight, efficient and easy deployment, and simple structure [1]. These aerial systems are being applied in a wide range of practical applications, such as inspection [2,3], surveillance [4,5], search and rescue [6,7], cargo transportation [8], etc. Over the past few years, many improvements have been made in quadrotor UAV modeling and control.

The sensors’ noise and environmental disturbances cause motion in the UAV’s platform, impacting directly in the camera-based measurement, harming both the mission and the inspection process. An example of a recent application is the inspection of solar panel farms by UAVs [9]. This automated monitoring practice provides rapid maintenance service, fast and efficient failure detection, extending the service life and performance [9]. UAVs can suffer from environmental disturbances, such as wind, magnetic fields, or uncertain disturbance effects, such as sensor noise or payload during inspections. In this sense, this aircraft must have a reliable and robust control for attitude control and stabilization problems [10,11].

Note that a design of high-quality flight is a tough effort for quadrotor UAVs due to their nonlinear characteristics [12]. To improve the UAV flight performance, several researchers proposed advanced approaches for stabilizing the attitude and improving the UAV positions and tracking [13,14]. Most of these works are based on fuzzy logic [15,16], sliding-mode controller (SMC) [17], neural network-based control [18], backstepping-based adaptive control [19], and a linear quadratic regulator (LQR) [20], among others.

Proportional-integral-derivative (PID) controllers have a simple structure. However, the nonlinear characteristics make the design and tuning of the PID complex. Furthermore, it cannot ensure closed-loop performance in different conditions of flight. Fuzzy controllers applied to UAVs undoubtedly have some advantages in performance evaluation compared to PID controllers, such as dynamic property and steady-state error [21]. In the work of [22], the authors used the Takagi–Sugeno (TS) technique, along with linear parallel distributed compensation for vehicle stabilization. Ref. [23] proposed the utilization of the Mamdani-type fuzzy control algorithm for attitude and altitude control. The authors of [16] proposed a Fuzzy-type PID hybrid approach to improve the attitude controller by adjusting the PID gains through fuzzy logic. PIDs are robust and widely used approaches for controlling and stabilizing robotic systems. The challenge arises in the gain values of the PID scheme to fit the robot in a different operational situation. For instance, in [24], the authors applied a self-tuned PID control in a quadrotor UAV to overcome the external disturbances. The authors of [25] used a hybrid PID control strategy to deal with wind disturbances. Many methods have been proposed for PID gain scheduling. For instance, the authors of [26] proposed an optimal gain scheduling backstepping controller based on the Particle Swarm Optimization (PSO) algorithm. Another scheduling approach was proposed in [27], where the authors proposed a single neural adaptive PID (SNA-PID) for rotor speed control. The use of a fuzzy controller can improve the system’s robustness and adaptability [28]. According to [29], the fuzzy-scheduling approach enables the possibility of having variable gains over time that can be more suitable and robust for uncertainties, disturbances, and possible faults. A systematic approach to tune these design parameters is an interesting task.

Scheduling the PID gain can improve the UAV in-flight performance and change the flight characteristics according to the performed task, as shown in [10,30,31,32]. This is related to the different requirements presented by different flight stages or applications. For example, moving the UAV in a given direction in an inspection application could generate a collision. In order to prevent this, it may be desired that the UAV prioritizes the control in this direction, allowing, for example, a more significant error in the opposed direction in case of a wind gust. As an example of different flight requirements tied to the flight stage, the ground effect can impact controller performance during take-off. Thus, an additional configuration can be useful concerning the distance from the soil.

Another example is the case of a UAV carrying a load that can move or changes weight during flight, such as carrying a cable [33]. I could be desired that, in sudden load change, the PID gain is modified to allow a response that avoids a crash. In such cases, permitting more significant position errors while focusing the motor power on maintaining height and stability is desired. Complex controller implementations emulate safety characteristics by enhancing control performance. In [34], the authors used an L1 controller to improve response to disturbances. A similar methodology was implemented in [35] by using an active disturbance rejection control (ADRC) controller. Both methods can be efficient by improving the response to disturbance. However, in safe critical conditions, only improving control performance may not be enough and it may be desirable to allow different margins of errors in distinct control axes.

There are already a few works that have tried to introduce in-flight features by using fuzzy logic. In [36], the authors applied a fuzzy logic to introduce safety aspects to landing. The authors replaced the PID controller with a fuzzy logic controller during the landing stage in their work. This strategy is functional but more complex than adjusting the gains to accommodate the differences. The same is true for [31], where the authors employed fuzzy to try to reduce tracking errors. However, their work relied only on simulations for the validation process. Ref. [37] proposed the use of an adaptive PID controller for fault-tolerant control and a fuzzy inference scheme for real-time tuning of PID controller gains. In the work of [38], the authors evaluated the performance between the hybrid fuzzy logic PD controller and classical PD controller. They used a real system to perform the tests. The results indicated that the fuzzy PD controller is more suitable for non-linear systems. Ref. [39] evaluated the stabilization performance of the quadrotor by using a classical PID against the fuzzy logic controller. The outcomes indicated that the fuzzy logic presented a faster response. In [40], the authors used a fuzzy system, tuning gains from the PID controller to stabilize the quadrotor to control the attitude and to track the desired trajectory. The inputs of the fuzzy controller are the speed required for the distance between the current position of the UAV and the reference point. The authors evaluated their methodology in a quadrotor in a MATLAB/Simulink environment.

Despite the many Fuzzy implementations in the literature, there is still space to propose methods to schedule the changes to improve UAV performance according to application needs. In this work, the authors present two fuzzy gain schedulers to evaluate the use of fuzzy logic to introduce this effect. The first controller changes the altitude controller gains as the altitude error changes. The second controller adjusts the position controller gains to reduce its action as the height error. Note that the features added by the second fuzzy gain scheduler, where the altitude error is used to control the position performance, are not present in other implementations [16,29,32,37,40,41,42,43,44,45]. This fuzzy controller allows changing performance following the UAV application requirements.

### Main Contributions

The main contribution of this research work is the proposition of a novel strategy based on a fuzzy-gain scheduling mechanism to adjust the PID controller to stabilize both position and altitude control. This control strategy must be effective, simple, and robust to uncertainties and external disturbances. The control parameters of the PID control structure are tuned and scheduled based on the fuzzy logic rules. The proposed strategy results were compared with a conventional PID controller. The contributions can be summarized as follows:

- Proposition of a novel PID-gain schedule through the use of a fuzzy logic scheme to stabilize the position and altitude controller of a UAV;
- Devise a strategy to tune Fuzzy PID controllers, considering environmental conditions.
- Present a testing solution that can be embedded on UAV companion computers using ROS;
- Evaluate the proposed strategy in simulated and real environments.

The rest of this paper is organized as follows. Section 2 formulates the kinematics and dynamics model of quadrotors. In Section 3, we describe the design of the fuzzy-gain scheduling mechanism to adjust the PID controller to address the position and altitude stabilization. Section 4 gives the proposed control performance in both simulation and real-world scenarios. Section 5 presents the concluding remarks and also the future works from this manuscript.

## 2. Quadrotor Modeling

**Figure 1:**
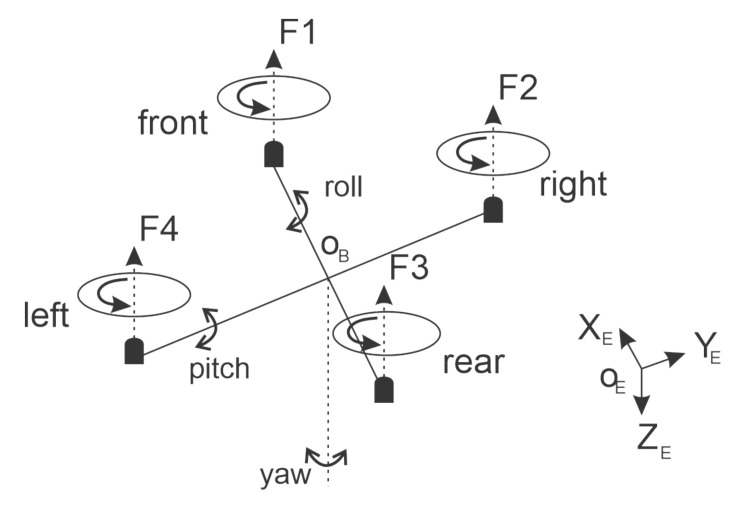
Quadrotor Mechanical structure.

The quadrotor UAV has four rotors attached to its structure, moving in hover, take-off, and landing positions by changing the speed of the rotor. This kind of system presents a stable yaw angle rotation that is represented in Figure 1. Note that F1, F2, F3, and F4 represent the four rotors. The center of the UAV is represented by $O_{b}$ , and ϕ is the roll Euler angle, θ is the pitch, and ψ is the yaw.

These Euler angles can be used as inputs on the forward kinematics equations to calculate the world-to-frame conversion matrix, which, based on the estimated values of roll, pitch, yaw, and velocity at each moment, can estimate the actual UAV position. Equation (1) gives this process calculation.

(1) $$R\psi ,\theta ,\phi =\left[\begin{array}{ccc} \cos(\psi ) & -\sin(\psi ) & 0\\ \sin(\psi ) & \cos(\psi ) & 0\\ 0 & 0 & 1 \end{array}\right]\cdot \left[\begin{array}{ccc} \cos(\theta ) & 0 & \sin(\theta )\\ 0 & 1 & 0\\ -\sin(\theta ) & 0 & \cos(\theta ) \end{array}\right]\cdot \left[\begin{array}{ccc} 1 & 0 & 0\\ 0 & \cos(\phi ) & -\sin(\phi )\\ 0 & \sin(\phi ) & \cos(\phi ) \end{array}\right]$$

The UAV body frame axes mathematical model is presented in Equation (2). Authors refer to [46] for this type of model limitation and characteristic. This equation uses auxiliary variables, which are Δ=cos(ϕ)sin(θ)cos(ψ) and Γ=cos(ϕ)sin(θ)sin(ψ) .

(2) $$\left[\begin{array}{c} \ddot{\phi }\\ \ddot{\theta }\\ \ddot{\psi }\\ \ddot{z}\\ \ddot{x}\\ \ddot{y} \end{array}\right]=\left[\begin{array}{c} \frac{I_{yy}-I_{zz}}{I_{zz}}\dot{\theta }\dot{\psi }+\theta \frac{J_{r}}{I_{xx}}\Omega_{r}+\frac{l}{I_{xx}}U_{2}\\ \frac{I_{zz}-I_{zz}}{I_{yy}}\dot{\phi }\dot{\psi }+\phi \frac{J_{r}}{I_{yy}}\Omega_{r}+\frac{l}{I_{yy}}U_{3}\\ \frac{I_{xx}-I_{yy}}{I_{zz}}\dot{\theta }\dot{\phi }+\frac{l}{I_{zz}}U_{4}\\ g-\frac{\cos\left(\phi \right)\cos\left(\theta \right)U_{1}}{m}\\ \frac{U_{1}}{m}\left(\Delta +\sin\left(\phi \right)\sin\left(\psi \right)\right)\\ \frac{U_{1}}{m}\left(\Gamma +\sin\left(\phi \right)\cos\left(\psi \right)\right) \end{array}\right]=\left[\begin{array}{c} \xi_{\phi }\left(t\right)\\ \xi_{\theta }\left(t\right)\\ \xi_{\psi }\left(t\right)\\ \xi_{h}\left(t\right)\\ 0\\ 0 \end{array}\right]$$

where ϕ is the roll Euler angle, θ is the pitch, and ψ is the yaw. The thrust and the actuation torques responsible for the roll, pitch, and yaw movements are represented by $U_{i}|\left(i=1,\ldots ,4\right)$ variables. Moreover, $\Omega_{r}$ is the engines residual angular speed. The variables $\xi_{\left(\phi ,\theta ,\psi ,h\right)}$ represent the disturbances that respectively affect the dynamics of the respective Euler angles.

One strategy to combine these variables is to use the Motor Mixing Algorithm. It is also possible to analyze how the $U_{i}$ inputs are related to the angular speeds of the rotors $\Omega_{\left(1,2,3,4\right)}$ , as shown in Equation (3). $U_{1}$ is the thrust equivalence force, $U_{2}$ represents a rotational force on the roll axis, $U_{3}$ represents a rotational force on the pitch axis, and $U_{4}$ represents a rotational force on the yaw axis. Note that $b(Ns^{2})$ is the impulse coefficient and $d(Nms^{2})$ is the drag coefficient.

(3) $$\left[\begin{array}{c} U_{1}\\ U_{2}\\ U_{3}\\ U_{4}\\ \Omega_{r} \end{array}\right]=\left[\begin{array}{c} b(\Omega_{1}^{2}+\Omega_{2}^{2}+\Omega_{3}^{2}+\Omega_{4}^{2})\\ b(-\Omega_{2}^{2}+\Omega_{4}^{2})\\ b(\Omega_{1}^{2}-\Omega_{3}^{2})\\ d(-\Omega_{1}^{2}+\Omega_{2}^{2}-\Omega_{3}^{2}+\Omega_{4}^{2})\\ -\Omega_{1}+\Omega_{2}-\Omega_{3}+\Omega_{4} \end{array}\right]$$

The fuzzy-gain scheduling technique is used to adjust the PID gains for both position and altitude controllers, reducing the UAV quadrotor error dynamics and improving the performance and robustness.

## 3. Control Strategy

**Figure 2:**
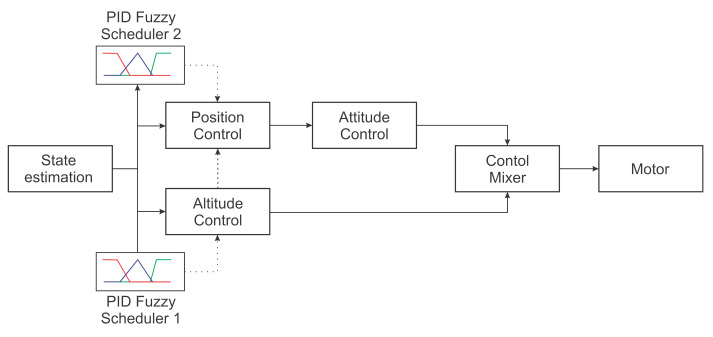
Proposed methodology.

This paper used a classic UAV control system composed of different stages to control the UAV position and orientation. Those stages were position, orientation, and altitude control. This model assumed that the full UAV state was known. Each PID controller was tuned using classical methods once the full UAV model was known. After that, a fuzzy scheduler was applied to increase or decrease the gain of the altitude control and the position control, allowing the controller to adjust to environmental changes. A simplified diagram of different control stages is illustrated in Figure 2.

**Figure 3:**
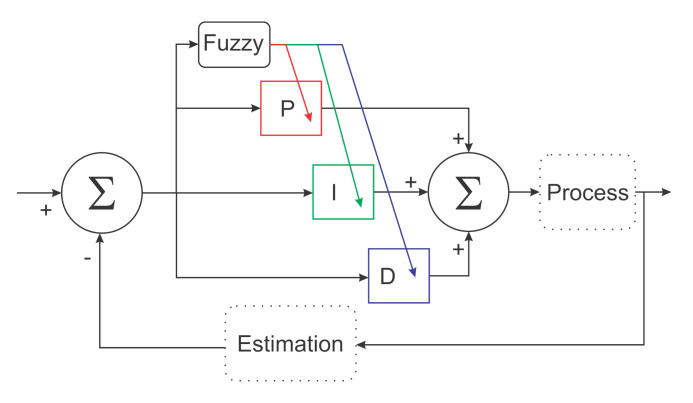
Fuzzy logic implementation.

Note in the diagram that the PID Fuzzy controllers were independent. Because of this, the authors could test the first controller separately and then evaluate them together. As these controllers were independent, the authors first worked on the altitude controller to produce an adequate response to the altitude error by adjusting the ideal controller for high and low altitude error environments. Once the altitude controller performance was within the desired values, the authors proceeded to the next step: designing the position controller and fuzzy scheduling for this controller. Each fuzzy implementation had one different objective related to the variable being controlled and its requirements. Each fuzzy controller measured the variable errors to determine the scheduling mechanism. Figure 3 shows a representation of this process.

Differences between fuzzy schedules aim to improve aircraft performance and introduce safety characteristics. The primary variable is the aircraft’s height and stability in this sense. Next, once the aircraft is stable, controlling the yaw keeps the aircraft from spinning. Controlling the yaw is important because it is not possible to perform position control without a stable yaw. In the end, the last objective is to keep the aircraft from losing its desired position. If the aircraft has plenty of thrust available, this prioritization may not be relevant once enough power is available to control all these parameters. However, in situations where battery voltage may be low, in the presence of wind gusts or payload change, the power available may not be sufficient to provide a stable response for all these variables. These scenarios may be beneficial to reduce control input for the position, such as allowing more space for height and stability control.

If a good prioritization is performed in the presence of a continuous high load, the aircraft would first start losing position. Then, it would start spinning to lose height at the end. This is an example of one implementation that can be extended to other applications, as already suggested in the introduction. Based on these, the fuzzy scheduler designed in this work evaluated a proposition to improve PID performance for height control and to prioritize height control over the position in determining cases. The strategy is a proof of concept because the more advanced and flexible implementation of fuzzy scheduling can be built using the same concepts. The authors detail the proposed implementation and its characteristics in the next subsections.

### 3.1. PID Tunning

The quadrotor altitude control design is well researched in many works [47,48]. In this work, a classic strategy was applied by linearizing the plant loop to obtain its reference model. This simplified model was used to provide reference performances for two operating conditions. The first condition was a very aggressive and fast controller that was used as the main control law. The condition was a smoother controller, and the fuzzy logic switched to this second control law following the observed errors. The plant model obtained is shown in Equation (4).

(4) $$\frac{0.000198\;\cdot z+0.000198}{z^{2}-2\cdot z+1}$$

The two control laws were obtained using the frequency response method to obtain a stable controller and good response times. The parameters obtained are shown in Table 1.

Regarding the parameters of Table 1, it is possible to quantify the performances, as presented in Table 2. Notice that the first control law had a slower rise time than a fast response at the cost of a larger overshoot. The second was smoother but took a little longer to respond.

**Figure 4:**
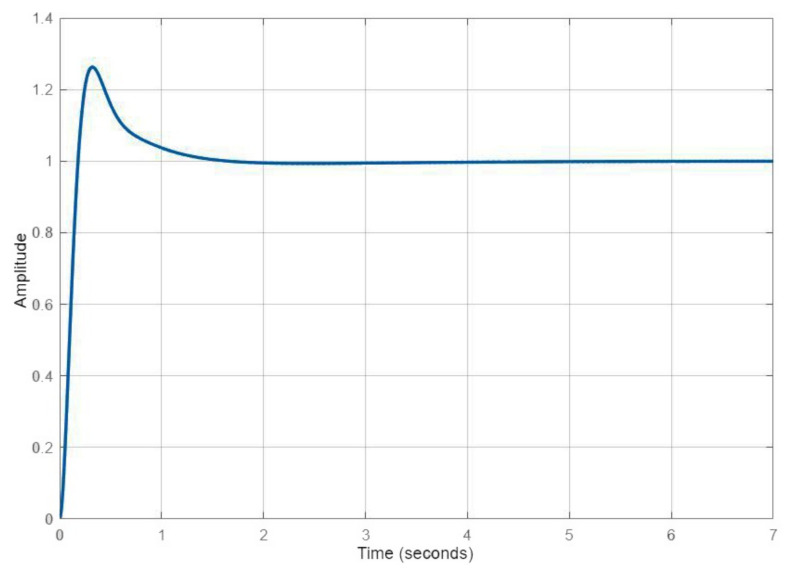
Altitude response to the first control law.

**Figure 5:**
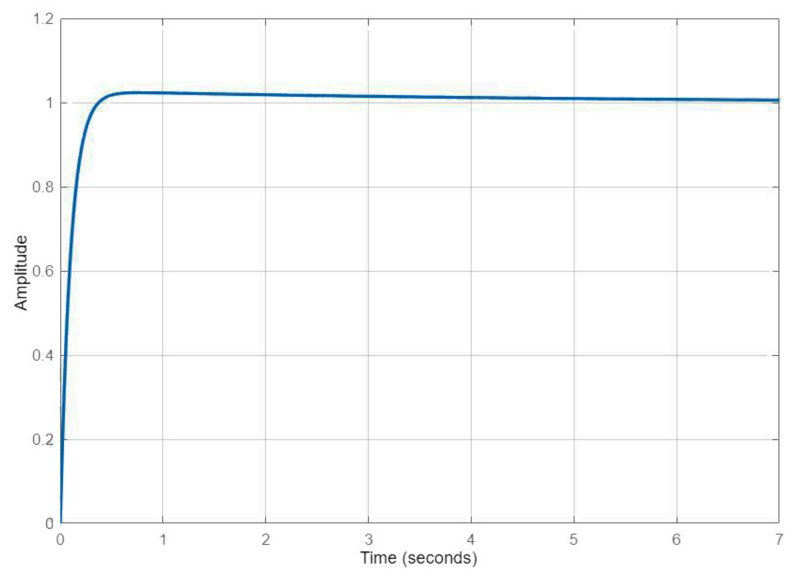
Altitude response to the second control law.

In Figure 4 and Figure 5, one can observe the qualitative response related to each of these control laws. A faster response meant that the UAV could respond to any environmental change faster. In order to avoid the overshoot, the fuzzy logic moderated the gains when closer to the target. Additionally, integral control was only added when the error was low enough to require steady-state compensation.

Once the altitude controller was set, the position controller used a similar strategy. The only differences was that a PD controller was used, and the first control law was configured to produce a good and robust response. The second control law produces a slow response that was used when the altitude error was very high. Table 3 shows the gains for the position control.

### 3.2. Fuzzy Gain Scheduler for Altitude Controller

For this controller and fuzzy scheduler, a simple strategy was applied. The P control gain was set to produce an overshoot response. In this configuration, the aircraft promptly responded to any change in the error. The fuzzy scheduler was responsible for moderating this gain when the target was closer, thus removing the overshoot behavior.

**Figure 6:**
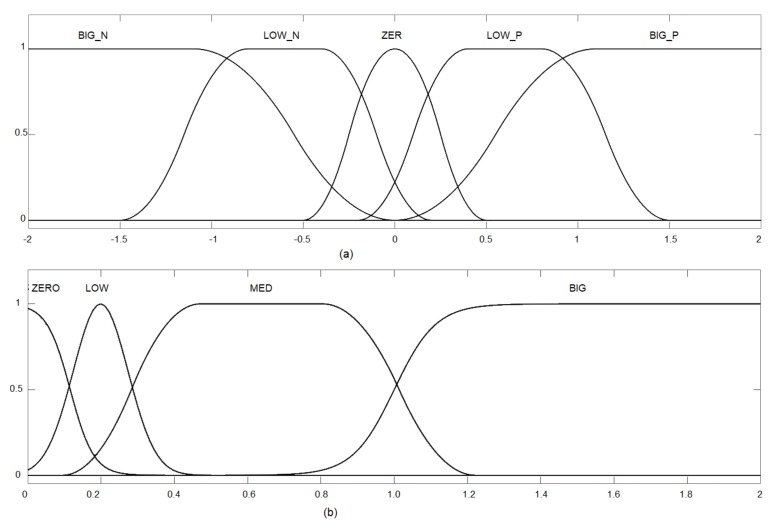
Input fuzzification: (**a**) Altitude error; and (**b**) Derivative of altitude error.

**Figure 7:**
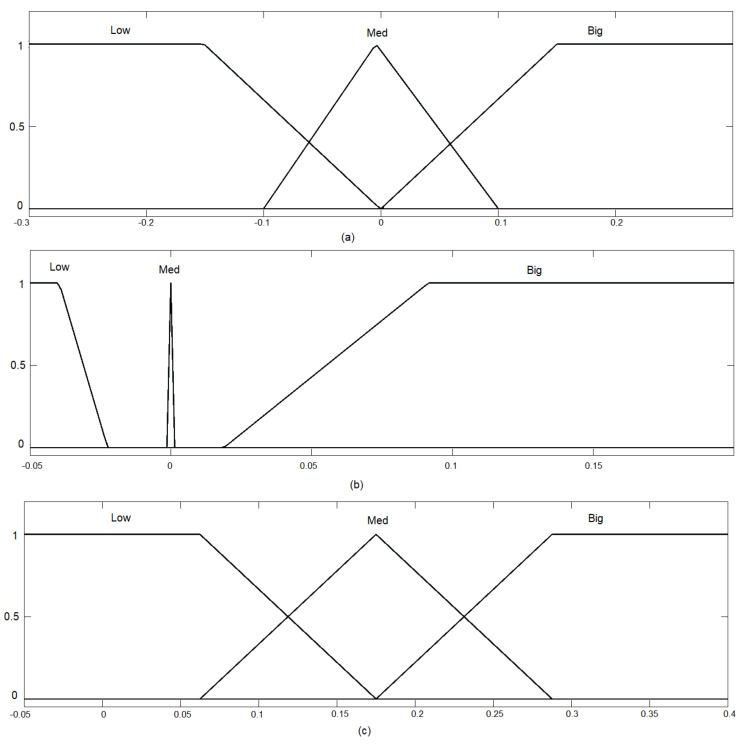
Output defuzzification: (**a**) Delta-P; (**b**) Delta-I; and (**c**) Delta-D.

The integral control signal was only applied when both error and error derivatives were low, meaning that the UAV was closer to the target. Otherwise, the integral action was not used. Figure 6 gives the input fuzzification, and Figure 7 presents the output defuzzification. Note that the inputs were the X-error (Figure 6a) and its time derivative (Figure 6b).

In Figure 7, the outputs are presented for each control effort of the PID controller. The values in the output represent the changes in the control law in effect, i.e., how much the PID gain was moderated or increased.

The idea behind the shapes selected for the input rules was to produce a selective transition between control laws as the error level increased. The output smoothly increased the PD gains to still and somewhat stable values as the error increased. However, the integrative error $K_{I}$ was only applied when small errors were present. This, along with anti-windup, ensured that steady-state errors were going to be corrected, but no instability would be produced.

**Figure 8:**
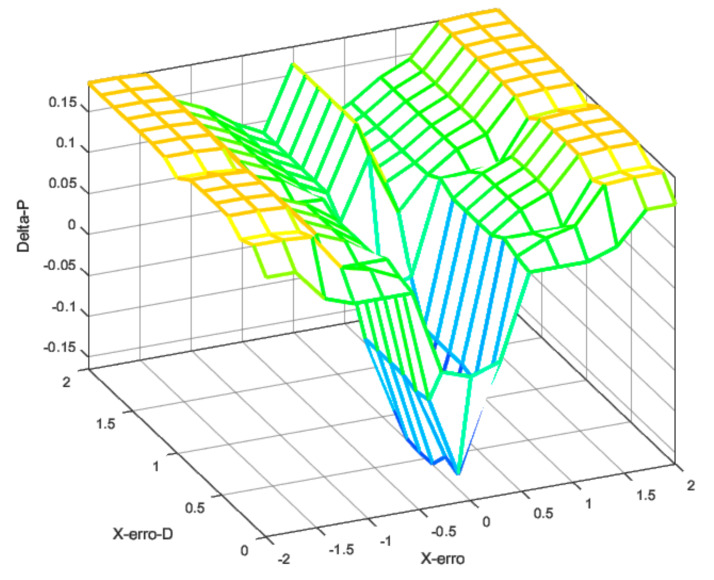
Fuzzy surface for proportional gain.

**Figure 9:**
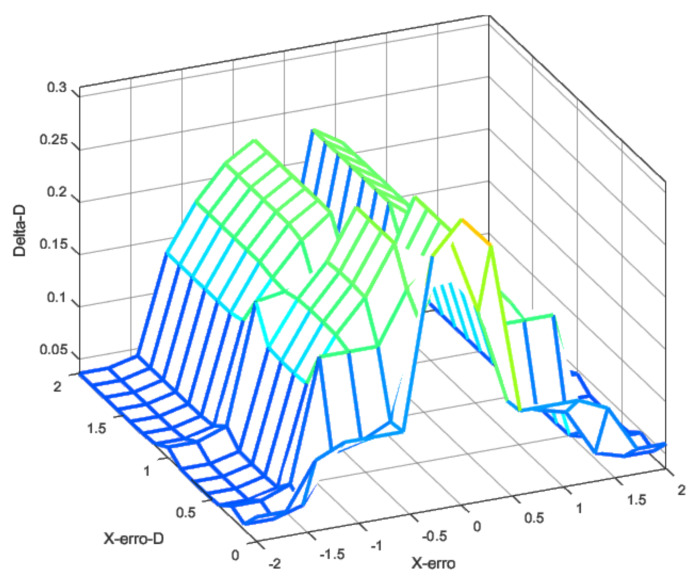
Fuzzy surface for derivative gain.

**Figure 10:**
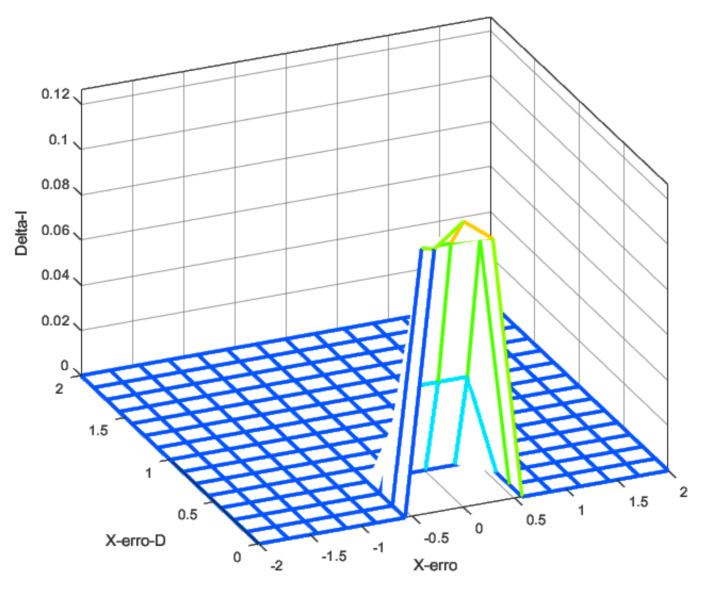
Fuzzy surface for integral gain.

Using the fuzzy rules and these fuzzy inputs, it was possible to design the controller to produce the transition between these two control laws according to the error and the error rate. Figure 8, Figure 9 and Figure 10 show the fuzzy surface generated by the rules applied using a Mamdani inference method [49]. The rules were built manually under the expected errors and were evaluated in different scenarios, such as response to the ramp, unit degree, and noisy input.

### 3.3. Fuzzy Gain Scheduler for Position Controller

The fuzzy applied in the position controller used a different strategy; a robust and fast position controller during normal operation. However, when the altitude error became too great, the gains were reduced to prioritize power to the altitude controller. The concept behind this strategy was to prevent the position controller from saturating the control signal in case of sudden altitude disturbances. In real UAVs, further levels would be required to produce a behavior that prioritizes height control first, then yaw control, stabilization, and at last, the position control. The idea behind these steps was to ensure that the UAV could continue flight in extreme conditions, such as load change, wind, and others. Only the interaction between the altitude and position control was evaluated in this work.

**Figure 11:**
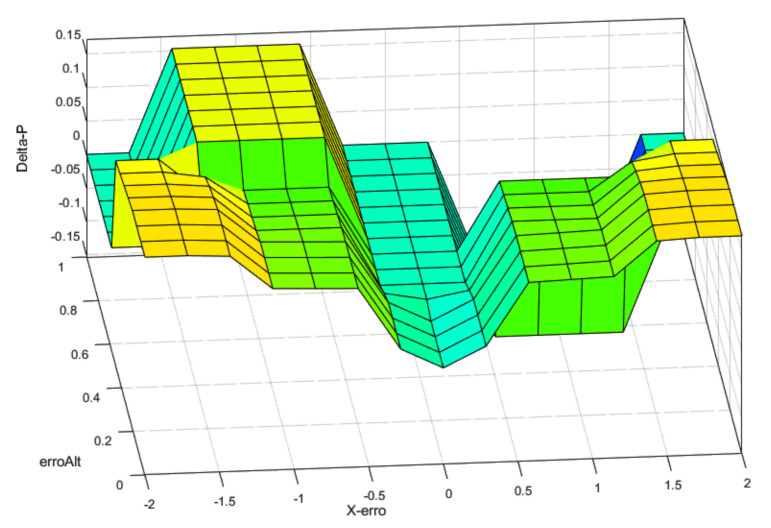
Fuzzy surface for position control-proportional gain.

Figure 11 shows the control surface designed for this controller. As altitude errors increased, the surface behavior changed, moderating the controller. This meant that, as the position error increased, the controller got a boost in the gains. However, the altitude error moderated the PD gains as the error increased. The same behavior was also implemented for the derivative control law.

## 4. Results and Discussion

**Figure 12:**
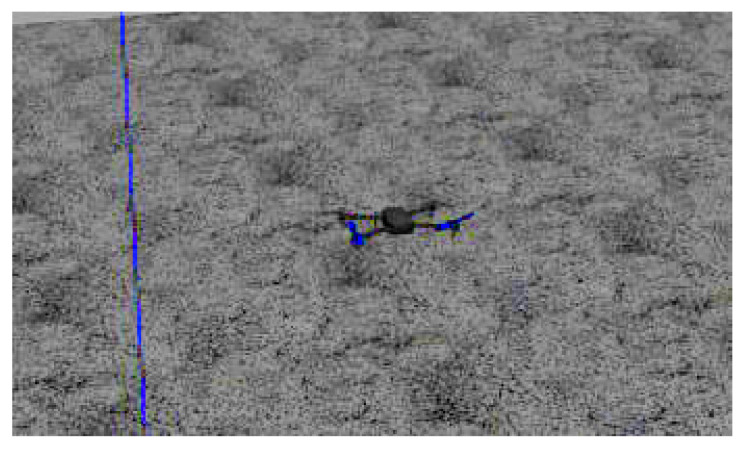
UAV used in the simulation tests.

For the simulation tests, a virtual world environment was created by using GAZEBO/ROS software platforms. Figure 12 illustrates the UAV 3DR IRIS used in the simulations. The GCS ran the operating system Ubuntu 18.04.4 LTS 64 bits, and it had an Intel Core i7-5500U CPU @ 2.40GHz x 4, Intel HD Graphics 5500 (Broadwell GT2) and a 15.6 Gb of memory. The desired position or speed set point was applied to the PX4 flight controller board, creating a simple and efficient external control loop.

The results section is divided into three parts. First, the authors evaluate the fuzzy schedule control usage to improve the performance of an altitude controller in a simulated environment. The different gains used are shown in Section 3.1. This step produced the effect of increasing the controller effort in response to larger errors while still allowing a smooth response. The authors evaluated the combined position and altitude gain, scheduling in the simulated environment from this step. This idea reduced the position controller effort when a high altitude error was observed while still producing an adequate response otherwise. At last, the height control scheduling was evaluated in a practical scenario as a control experiment to validate the first simulation. The evaluation of a complete architecture in a realistic scenario is proposed for future works.

### 4.1. Results for Fuzzy Gain Scheduler for Height Controller

**Figure 13:**
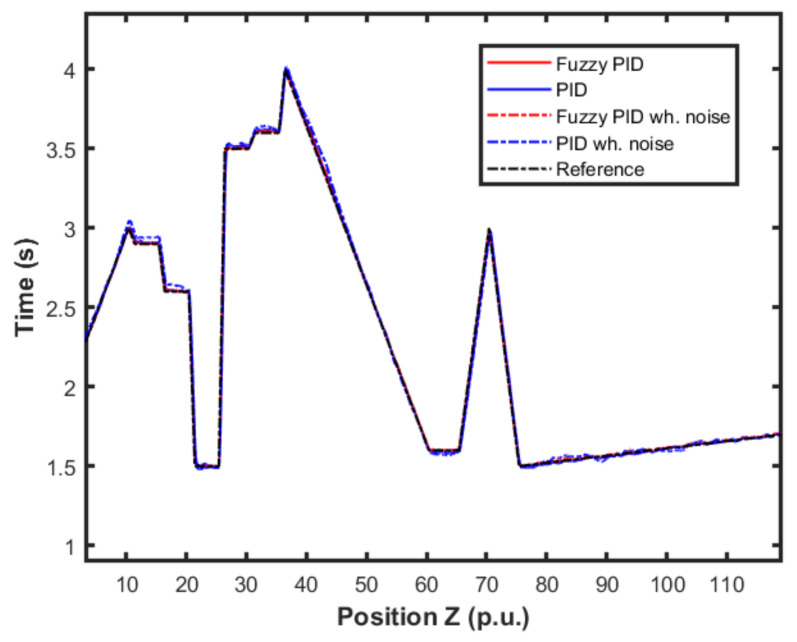
Comparison of the proposed method and PID controller for altitude control.

In this first experiment, a mixed set of inputs was used to evaluate the controller performance when responding to the increase and decrease errors with different slopes. The performance for this mixed set of inputs summarizes the controller performances for the original and scheduling controller with and without noise. The result for the altitude control using the fuzzy gain scheduling strategy is shown in Figure 13. Note that distance units here were standardized in the per unit (p.u.) system. The proposed scheme and the classical PID control were both applied to the system for comparison purposes, regarding two different scenarios: (i) in the absence of measurement noise and (ii) in the presence of 10% of measurement white noise with constant probability distribution.

**Figure 14:**
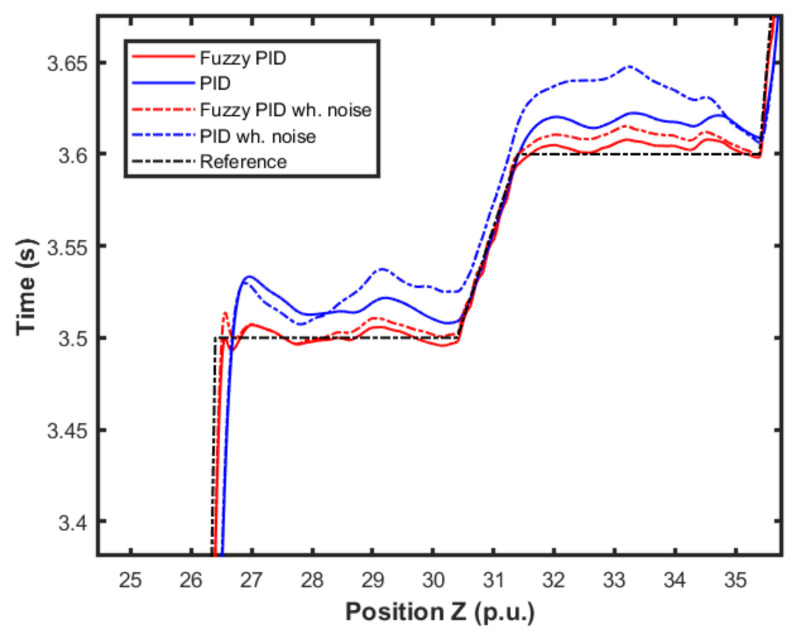
Detail of comparison of the proposed method and PID controller for altitude control.

Figure 14 presents an approximation of Figure 13. It was possible to observe more closely the performances of each controller and scenario. We were able to notice the differences in performance for each controller in relation to the reference.

**Figure 15:**
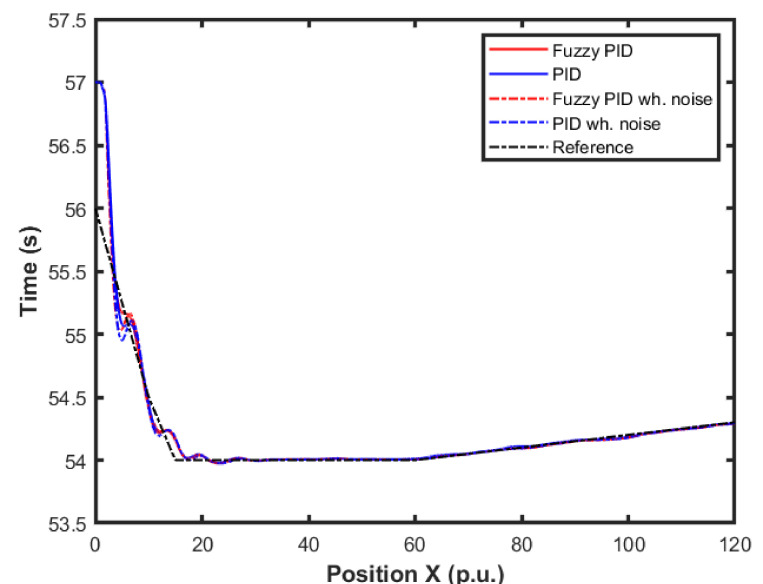
Comparison of the proposed method among others for position control in the X-axis.

**Figure 16:**
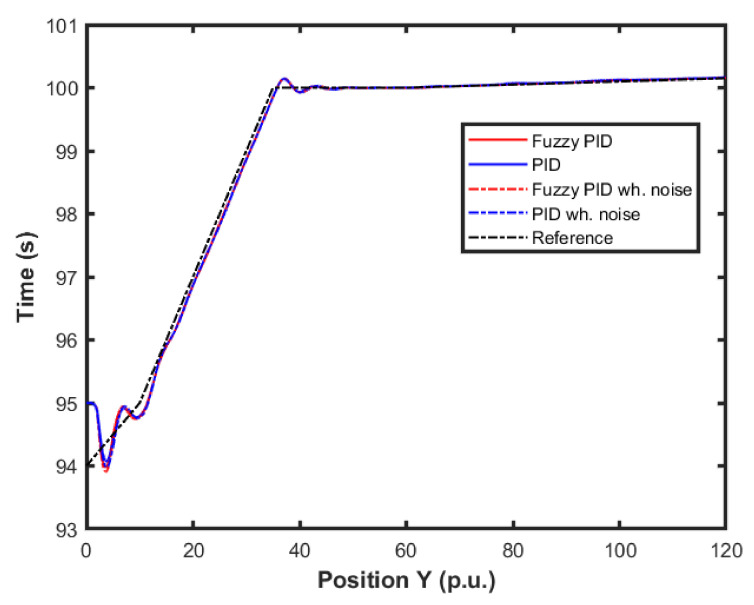
Comparison of the proposed method among others for position control in the Y-axis.

Figure 15 and Figure 16 show the performance of the proposed fuzzy gain scheduler for position control along the z-, x-, and y-axis, respectively. This kind of result can help us to understand the importance of adaptive controllers for UAVs. In Figure 14, it is possible to note that the fuzzy gains presented a slightly smaller error in the z-axis when compared to the other control strategies. Additionally, during stabilization, the output obtained with the proposed strategy oscillated less than the results obtained with the other controllers, thus achieving the positioning objective in a more well-behaved profile.

**Figure 17:**
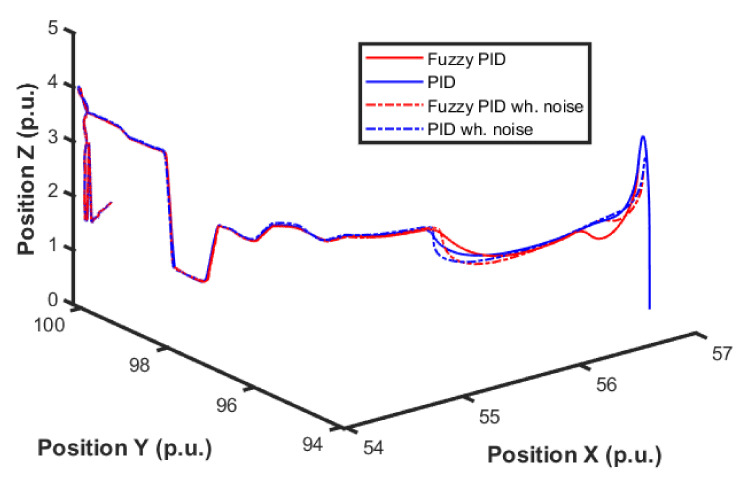
3D visualization for the comparison of the proposed method among other approaches for position control.

For better visualization, Figure 17 shows a 3D profile of the results for comparisons among other control approaches. It is possible to observe the good performance, in terms of smaller error amplitude, of the PID control that had the gains estimated by the proposed fuzzy scheduling approach.

### 4.2. Results for Fuzzy Gain Scheduler for Position Controller

**Figure 18:**
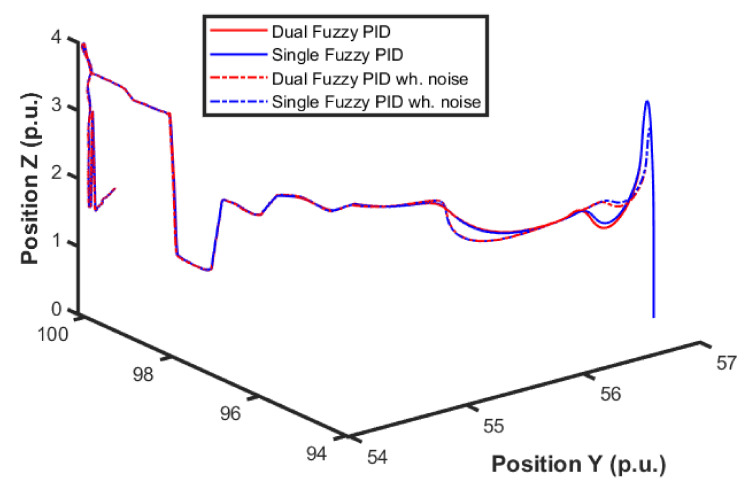
UAV 3D path during simulations.

The next series of simulations consisted of applying this strategy using the previous fuzzy controller alone and using both (i.e., altitude and position) controllers. In this test, the position control error became relevant, and the entire 3D path is shown in Figure 18. Four different controllers were analyzed, which are PID without noise, PID with noise combined fuzzy without noise, and combined fuzzy with noise. As the position controller prioritized altitude, a minor overall error was expected.

**Figure 19:**
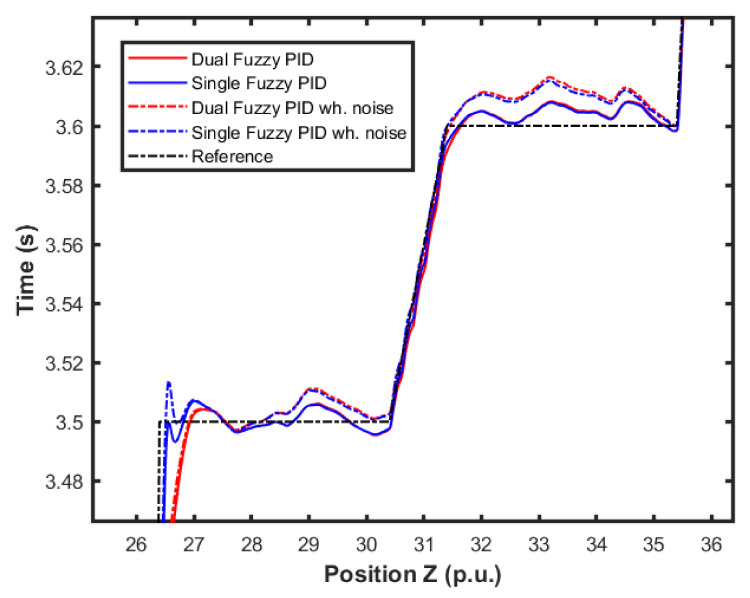
UAV height response details.

By looking at the detail of the height response at the end of a step (shown in Figure 19), it is possible to observe the combination of these effects of the controllers. Note that the error in the position controller was only affected when the altitude error was higher. Quantitatively, the error was 2% lower.

### 4.3. Experimental Results

**Figure 20:**
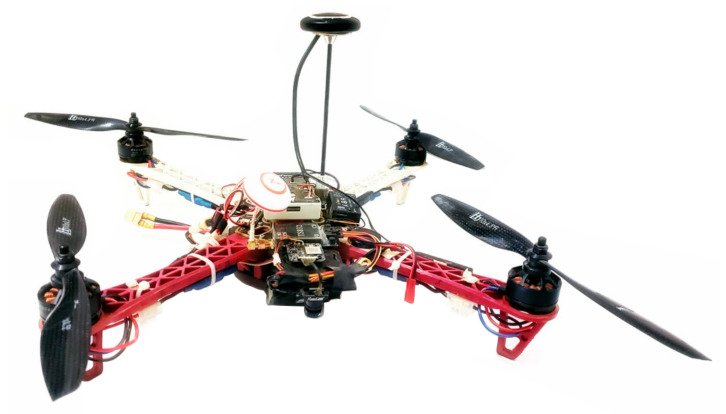
Quadrotor used in the experimental tests.

In order to test the performance of the proposed fuzzy gains-scheduling for the PID control in a real-world scenario, the quadrotor used in Figure 20 was used. This UAV was embedded with a Pixhawk 2.4.8 32-bit flight controller unit (FCU), with an Ardupilot firmware, a NEO-8M GPS, and a companion computer. The FCU of the quadrotor was compatible with the network interface for a Robotic Operating System (ROS) and the MavLink communication protocol for a telemetry and offboard controller. The MAVROS interface allowed real-time information access.

This experiment evaluated both the position and altitude controller. The original gains were obtained from standard PID gains.

#### 4.3.1. Altitude Control

In this first experiment, the UAV was subject to a fast input change in the altitude stick, and the position response was measured, simulating a step response. The proportional gain was changed to modify aircraft responsiveness, and the process was repeated. The proportional gain was kept fixed, while derivative and integral gains were adjusted. Note that this practical strategy was used to avoid any possible modeling issues in the PID adjustment.

**Figure 21:**
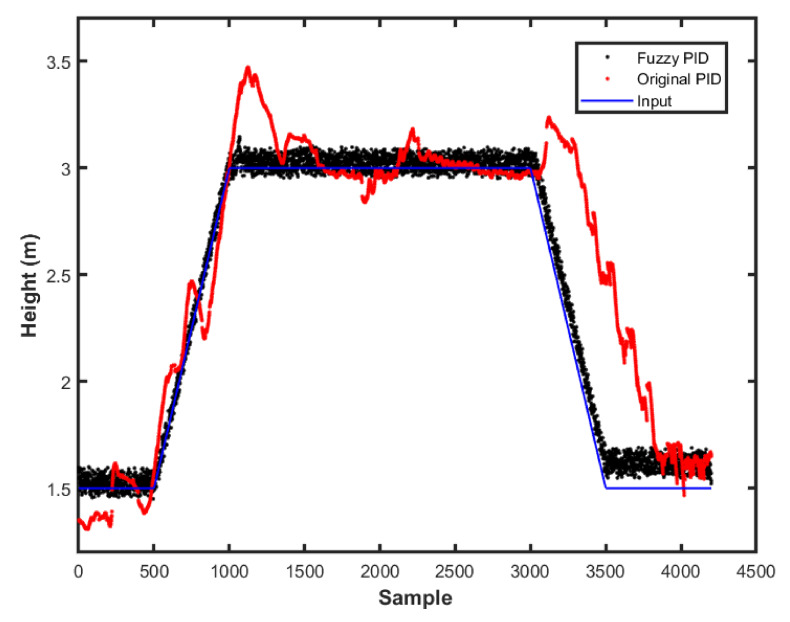
Height response for the practical experiment.

The fuzzy-PID and PID gains were selected using the same strategy as in the simulations, using the values obtained in the previous phase. The UAV was submitted to a few inputs to simulate step response and evaluate the methods. Figure 21 presents the UAV height during the first experiment.

The behavior observed in the practical experiment was that the fuzzy scheme produced a higher noise than the original controller. This may be related to the increased gains used and their scheduling. It could also be due to the sensors amplified noise in the controller. However, the height tracking was more noticeable, being closer to the reference. We could also use this data to estimate the controller performance parameters, as seen in Table 4.

This work used a fuzzy scheme to adjust the altitude and position controller following the environmental changes. Each fuzzy controller measured the variable error to determine the scheduling approach. The first fuzzy-gain scheduler, applied to the altitude controller, was based on the output error and time derivative. This process is common in most works of literature. However, the second fuzzy-gain scheduler for the position controller was based on the position and altitude errors, which is different from other works. Note that the PID-gain was chosen through frequency response. However, other methods, such as the Ziegler–Nichols ultimate gain and oscillation period method, could be used.

#### 4.3.2. Control on Critical Condition

In this second experiment, the authors intended to evaluate the control scheme performance in a critical condition. This meant that the authors commanded the UAV to a given height and then added height to simulate a sudden increase in power requirements from the motors, thus allowing the fuzzy scheme to prioritize power to the altitude control.

**Figure 22:**
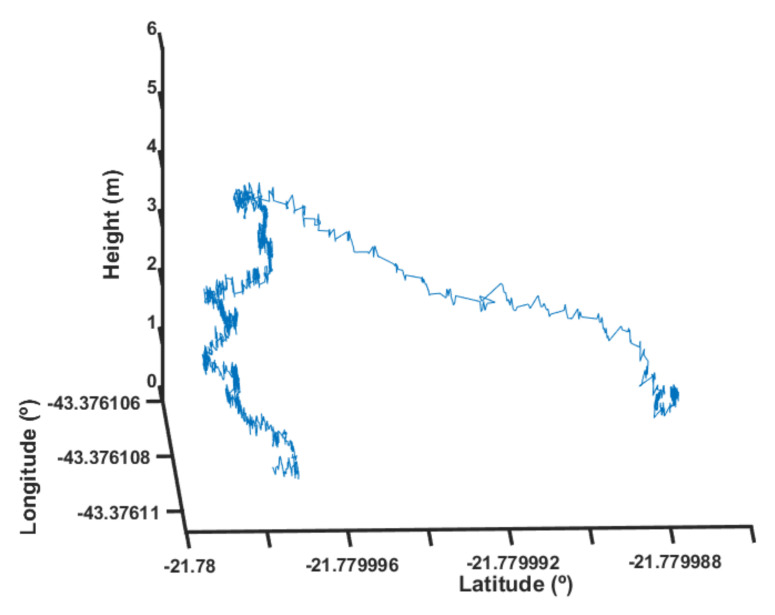
Height response without fuzzy scheduling.

The steps taken were the first take-off of the UAV and finding a stable position control while hovering at 1 m. Then, the authors applied a 5-m step with a load attached to a 4-m cable. This situation increased the UAV weight to 95% of its designed load. In Figure 22, one can observe the experimental results for a condition where the fuzzy scheduling was not present. One can notice that, during the few seconds as the load increase took effect, both the position and height control stopped operating properly.

It is important to note that the scale of the vertical axis (height) was different from the scale of the horizontal axes (latitude and longitude). The latitude axis of Figure 22, for example, had approximately 1 m of length.

**Figure 23:**
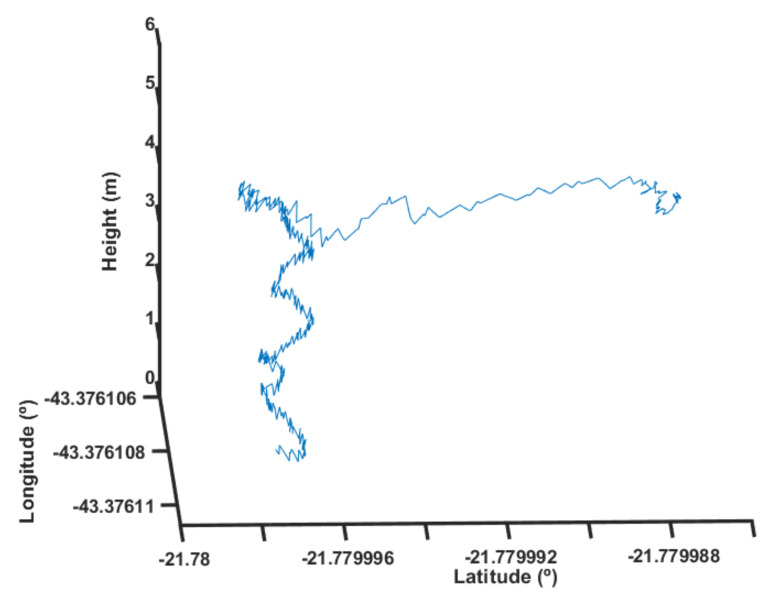
Height response with fuzzy scheduling.

Next, the authors used the same strategy but applied the fuzzy scheduling. This process was repeated five times to quantify the average response observed. Figure 23 shows the result for one of these tests with the fuzzy scheduling.

One can notice that the UAV was capable of maintaining a relatively stable position up to its designed height with a little overshoot. The overshoot is a possible sign of the position and height control interaction when subjected to the high disturbance caused by the load. Additionally, it is possible to notice that the position control lost reference very quickly. A little height loss was observed as the UAV could not maintain its altitude due to the motors’ thrust condition.

**Figure 24:**
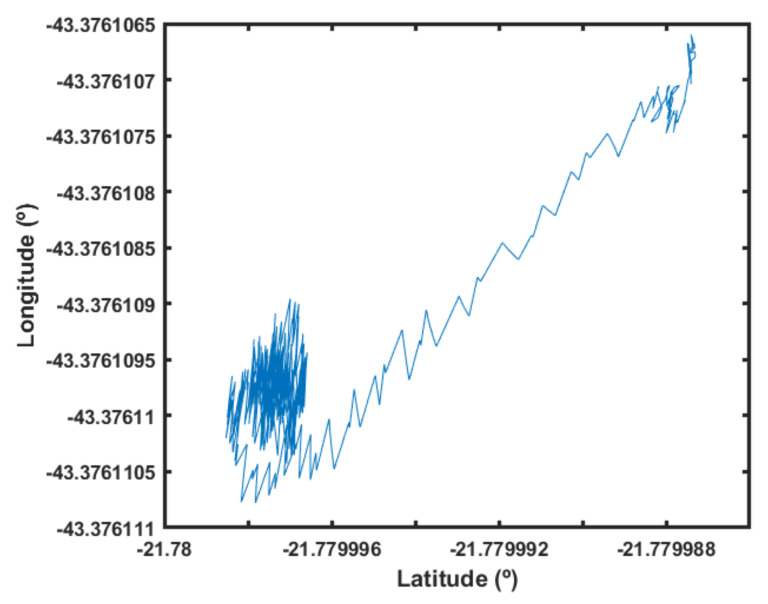
Position response.

This is easier to visualize through Figure 24, where the position response is isolated. Note that the positions were concentrated at a given area related to the wind disturbance and GPS errors. Suddenly, the position reference was lost, and the UAV started to deviate from its location.

To quantify the performance improvement in this condition, the height error in the last 500 samples was averaged. The fuzzy scheduler observed an average error of 0.765 m, while the average error was 2.038 m without it. The absolute improvement value was not very informative when the ground truth was not captured externally. However, this difference shows that the method can potentially change UAV flight behavior, adding different flight characteristics for different situations.

## 5. Conclusions and Future Work

The PID controller is a simple control scheme, widely applied in the literature. However, as a linear control strategy, it is observed that its performance suffers degradation when it is applied in linearized systems at different operating points. The sudden change in operating points is usually due to rapid changes in motion. In such a scenario, the gain schedule technique is an attractive solution to adjust this type of controller during changes in plant operating regions. In this sense, this work developed a fuzzy gain-scheduling PID controller for a quadrotor vehicle in order to control its position and altitude. The tests were performed both in a simulation environment and in a real quadrotor system. The obtained results for the trajectory tracking problem showed that the proposed control-plus-tuning strategy revealed a good performance compared to the classical PID results, even considering the presence of measurement noise.

The fuzzy logic has multiple parameters and instructions that impact directly in the processing speed. Therefore, this work opens the possibility of several improvements in terms of implementation. For instance, optimal algorithms could be used to improve the processing speed. Another potential interest is dealing with the vehicle payload changes, which usually occur in product delivery situations.

In terms of evaluation, this research work opens up several future possibilities. In future works, authors expect to implement a complete version of the PID scheduling method, capable of handling complex situations by prioritizing height first, stability pitch, roll, and third yaw second, and position control last. In future works, the authors also intend to test strategies to perform control in restricted spaces, where a few directions have more freedom of movement than others. For instance, inspections close to structures, where the UAV can’t move in the structure direction but can go freely in another way. The MATLAB/Simulink software was used for the proof of concept of the Fuzzy Logic algorithm. However, in future works, this should be embedded in the FCU of the UAV, avoiding delays in the information processing.

## Figures and Tables

**Table 1:** Gain for the altitude control law.

	Gain	
	**Control Law 1**	**Control Law 2**
P	1.7	0.16
I	0	0.009
D	0.6	0.6

**Table 2:** Performance of altitude controllers.

	Control Law 1	Control Law 2
Rise time	0.12	0.2
Setting time	1.0	1.6
Overshoot	20%	2%

**Table 3:** Gain for position control law.

	Gain	
	**Control Law 1**	**Control Law 2**
P	0.24	0.07
I	0	0
D	0.1	0.05

**Table 4:** Controller performance.

	Original	Fuzzy
Rise Time	0.1 s	0.1 s
Settling Time	0.1 s	0.2 s
Overshoot	13%	1%

## Data Availability

The source-codes are openly available in https://github.com/GeneralAdmin/FuzzyGainSchedulingUAV (accessed on 2 February 2022).

## Abbreviations

The following abbreviations are used in this manuscript:

MDPI	Multidisciplinary Digital Publishing Institute
PD	Proportional Derivative
PID	Proportional Integral Derivative
UAV	Unmanned Aerial Vehicle
ROS	Robotic Operating System
SMC	Sliding-Mode Controller
PSO	Particle Swarm Optimization
SNA-PID	Single Neural Adaptative PID
ADRC	Active Disturbance Rejection Control
